# Supplementary material for: Development of visual cortex in human neonates is selectively modified by postnatal experience
Source: eLife. 2022 Nov 18;11:e78733. doi: 10.7554/eLife.78733 (PMC9674344; doi:10.7554/eLife.78733)
Supplement: Figure 1—source data 1. [file elife-78733-fig1-data1.docx]

**Figure 1-source data 1.** The labels of 34 ROIs in the ventral cortex and 15 of the 34 ROIs (bolded) are in the ventral occipital temporal cortex.

| **Number** | | **Area Name** | **Area Description** |
| --- | --- | --- | --- |
| 1 | V1 | | Primary Visual Cortex |
| 2 | V2 | | Second Visual Area |
| 3 | V3 | | Third Visual Area |
| 4 | V4 | | Fourth Visual Area |
| **5** | **V8** | | **Eighth Visual Area** |
| 6 | V3CD | | Area V3CD |
| 7 | LO3 | | Area Lateral Occipital 3 |
| 8 | LO1 | | AreaLateral Occipital 1 |
| 9 | MT | | Middle Temporal Area |
| 10 | MST | | Medial Superior Temporal Area |
| **11** | **V4t** | | **Area V4t** |
| **12** | **LO2** | | **Area Lateral Occipital 2** |
| **13** | **FST** | | **Area FST** |
| **14** | **PIT** | | **Posterior InferoTemporal complex** |
| **15** | **PH** | | **Area PH** |
| **16** | **TE2p** | | **Area TE2 posterior** |
| **17** | **FFC** | | **Fusiform Face Complex** |
| **18** | **VVC** | | **Ventral Visual Complex** |
| **19** | **VMV3** | | **VentroMedial Visual Area3** |
| **20** | **VMV2** | | **VentroMedial Visual Area 2** |
| **21** | **VMV1** | | **VentroMedial Visual Area 1** |
| **22** | **PHA3** | | **ParaHippocampal Area 3** |
| **23** | **PHA2** | | **ParaHippocampal Area 2** |
| **24** | **PHA1** | | **ParaHippocampal Area 1** |
| 25 | PHT | | Area PHT |
| 26 | TE1p | | Area TE1 posterior |
| 27 | TE1m | | Area TE1 middle |
| 28 | TE1a | | Area TE1 anterior |
| 29 | TGd | | Area TG dorsal |
| 30 | TE2a | | Area TE2 anterior |
| 31 | TF | | Area TF |
| 32 | EC | | Entorhinal Cortex |
| 33 | PeEc | | Perirhinal Ectorhinal Cortex |
| 34 | TGv | | Area TG Ventral |

Note: the area names and area descriptions were identical to the original literature (Glasser et al., 2016).

**Reference**

Glasser MF, Coalson TS, Robinson EC, Hacker CD, Harwell J, Yacoub E, Ugurbil K, Andersson J, Beckmann CF, Jenkinson M, Smith SM, Van Essen DC. 2016. A multi-modal parcellation of human cerebral cortex. *Nature* **536**:171–178. doi:10.1038/nature18933
